# Formaldehyde

**Published:** 1897-07

**Authors:** 


### Abstracts and Translations.

### FORMALDEHYDE.

In the *Medical Chronicle* for December, 1896, Leech gives an interesting summary of the therapeutic possibilities of formaldehyde. Formic aldehyde ($CH_2O$) is produced when by means of a specially constructed lamp the vapor of methyl alcohol ($CH_3OH$) is passed over an incandescent platinum hood or mantle. The following formula represents the reaction: $CH_4O + O = CH_2O + H_2O$. For some time past a solution of formaldehyde in water of a strength of forty per cent. has been on the market under the name of formol or formalin.

Mosso and Paoletti find that formalin has a bacterial action almost equal to that of corrosive sublimate, while it is much less toxic. One part in 20,000 is sufficient to slow the ammoniacal fermentation of urine, and 1 in 4000 inhibits it altogether. Formalin hinders the coagulation of albumen by heat, but hastens the clotting of blood. It has little influence on the frog's heart, unless in solutions over one per cent. in strength. Very small doses, however, are sufficient to raise the blood-pressure and markedly affect respiration. Doses exceeding one cubic centimetre per kilo of body weight quickly cause death; doses of 0.1 cubic centimetre are poisonous if introduced into the circulation; and even smaller doses produce marked symptoms of irritation. A powerful action on the nervous system is shown, resulting in convulsions, analgesia, and lowering of temperature.

Formaldehyde has been found very useful in pathological work for hardening microscopic preparations and museum specimens. Orth has recently pointed out the value of formalin in this connection.

Several observers have experimented with formaldehyde in the disinfection of rooms. Some of the more recent papers are those of Roux, Trillat, Pfuhl, and Hebert. Opinion is divided as to the practical value of formaldehyde for this purpose.

Horton considers formalin particularly suitable for the disinfection of books, as the vapor is not detrimental in any way to them, while it is very rapid in its disinfectant action. The effect produced during the first fifteen minutes is practically as great as that after twenty-four hours' exposure. He found that in a closed space books can be thoroughly disinfected by using one cubic centimetre of commercial formalin to three hundred cubic centimetres of air.

Turning to the therapeutic uses of the drug, Schleich found that when a watery solution of gelatin is allowed to dry in formalin vapor the chemical characteristics of the gelatin are altered. It is no longer affected by hot or cold water, nor by acids or alkalies. Animal tissues, however, have the power of breaking up the combination and setting the formalin free. It was also found that when the formalin gelatin, ground to a fine powder and mixed with cultures of various forms of pathogenic bacteria, was introduced into animals the bacteria did not develop, and the wounds healed without trouble.

Schleich states that with this formalin gelatin powder every acute suppuration can be stopped in twenty-four hours, and wounds made to heal aseptically. He has used it in one hundred and twenty cases of acute suppurative processes, in ninety-three aseptic wounds, four compound fractures, and two deep scalp wounds. The wounds were only cleansed mechanically, and then thoroughly rubbed with the powder. In fresh wounds the powder formed with the blood a quite dry and firm scab in a few hours.

In cases of necrotic masses, in old ulcers, etc., the powder had very little effect, but it was found that it could be digested with a pepsin hydrochloric acid solution (5 parts of pepsin and 0.3 of hydrochloric acid in 100 parts of water). The formalin gelatin powder is dusted on the wound, and then covered with a dressing wet with the pepsin solution, and the digestive process keeps up a continuous supply of formaldehyde vapor for the wound. The powder is made by drying 500 grammes of purified and dissolved gelatin in the vapor of twenty-five drops of formalin.

Foote has recently published a paper giving an account of forty-five cases of suppurative wounds in which he has used Schleich's formalin gelatin. He concludes that formalin has some antiseptic action, but not so great as to render a suppurating wound sterile. It seemed to control the infection for two days, and if the character of the wound was such that this respite was enough to secure its closure, the result was perfect. If not, then whatever gain was made in the first two or three days was maintained, and the wound went on granulating from that point. This, however, is a distinct advance on the usual treatment. Another point in favor of the formalin gelatin is that it does away with the necessity of drainage. On the whole, Foote thinks the method marks a distinct advance in the treatment of suppuration, giving the most perfect results in those cases where the cellulitis is moderate and the pus abundant.

Alexander considers formaldehyde the "ideal germicide, deodorant, and antizymotic." He has used it in his practice for a year. He quotes De Buck and Vanderlinden as having used it successfully in one-half per cent. strength for washing hands and instruments, cleansing site of operation, and for rendering infected wounds, cavities, and sinuses antiseptic. Formalin does not spoil the edge of the knives, apparently not attacking metal at all. Dr. Alexander uses the pure forty per cent. formalin very successfully in chancroid and chancre, applying it locally, a single application being sufficient to cause the ulcer to heal rapidly. He found formalin solution a remedy for pruritus vulvæ when other drugs had failed. Four cases of diphtheria were treated with formalin and whiskey. The whiskey was given internally, and the atmosphere of the room impregnated with the vapor of formalin, direct application to the throat being also made with the formalin solution. He finds a spray of one-half per cent. valuable in hay fever, and a spray of one-per-cent. solution in whooping-cough. In ten cases of gonorrhœa he used a one-half-per-cent. solution, injected three times a day, with satisfactory results; he found the treatment free from the pain or irritation usually caused by the use of sublimate and other solutions.

Howland has treated six cases of gonorrhœa with formalin. In every case the gonococcus was found. He started with a five-per-cent. solution, but found this too strong. In the rest of the injections he used a one-half-per-cent. solution. For the first two or three days irrigations of one quart of hot formalin solution were given twice daily; afterwards once daily until the discharge ceased to contain the gonococci. No internal treatment was given except cathartic pills. All highly-seasoned food, alcohol, tea, and coffee were prohibited. The patients were advised to drink two to four quarts of pure water in the twenty-four hours. Dr. Howland noticed peculiar action of the irrigating fluid on the gonococci. They "shrivelled up" and lost their form.

De Smet claims good results from the use of formaldehyde in gonorrhœa in women. Sixty cases, some very obstinate, were cured. The vulva was washed with a 1 : 1000 solution, and the vagina douched through a speculum with a strong solution, varying from 2 : 1000 to 5 : 1000. If the uterine cavity and cervical canal were involved, some of the same solution was injected. When there is laceration of the cervix, tampons soaked in 1 : 1000 solution of formaldehyde are left for two or three hours in the vagina. When fungous endometritis is present the curette must be applied first. The applications give rise to no pain, and may be used daily, or every second day.

Lamarque had used formol in one-per-cent. solution for washing out the bladder and urethra, and in five-per-cent. solution for instillation. In acute gonorrhœa and in gonorrhœal cystitis he has not had encouraging results. In chronic gonorrhœa they have been better. He considers this treatment most successful in cases of tubercular cystitis. The only disadvantage is the pain caused by the drug, which, however, though intense, quickly ceases.

In ophthalmic practice formaldehyde has been used for some time. Valude, in May, 1893, made a communication on the subject to the Société Française d'Ophthalmologie.

Burnett has obtained excellent results in infecting ulcers of the cornea and in purulent conjunctivitis. Corneal ulcers may be touched with a solution of 1 : 200 or 1 : 500 every day. For general use as an antiseptic collyrium, a strength of 1 : 1000 or 1 : 2000 may be used, though the stronger of these sometimes causes a slight burning sensation.

Davidson finds one part of formalin in two thousand or three thousand of water the most serviceable strength of solution. When he tried it first in hypopyon ulcers, it was dropped into the affected eye three or four times daily, and it seemed of very little use, but on applying it freely every hour it acted very effectually. In abrasions of the cornea and in corneal ulcers, Dr. Davidson believes formalin will be of great value if applied freely and often.

Dr. Stephenson has found a solution of 1 : 2000 of service in muco-purulent and follicular inflammations of the conjunctiva when applied thrice a day to the everted lids. In trachoma it seems to have the power of reducing the amount of secretion.

Solis-Cohen has during the past year seen such good results in the treatment of tuberculosis of the larynx, alike in infiltrative, ulcerative, and vegetative cases, by means of formic aldehyde solutions, that he is tempted to believe that in this agent we have a means of treatment superior to any other that he has ever used. He uses the commercial formalin, diluting it to the strength required, which ranges from one-half to four per cent. of formic aldehyde,—that is, from one to ten per cent. of the commercial formalin, which contains 0.40 per cent. of formaldehyde. Before making the applications the parts should be thoroughly cocainized, otherwise the application to the mucous membranes causes an intense burning, stinging, and even strangling sensation.

The mode of application is similar to that employed with lactic acid. The parts are thoroughly rubbed with the formaldehyde solution after previous cleansing and cocainization. Beginning with the weakest solution, the strength is increased up to ten per cent. of the commercial formalin, which corresponds to four per cent. of pure formaldehyde. This is the strongest solution he has found it necessary to employ.

Pottevin has tried formic aldehyde for ringworm. The hair having been cut short, and the scalp cleansed, a compress of cotton-wool soaked in a two-per-cent. solution of formic aldehyde was applied to the affected parts, or, better still, to the whole scalp. The whole was then covered with an india-rubber cap, or piece of oiled silk, and left on for twenty-four hours, when a fresh application was made.

The results were not encouraging, as in most cases the remedy did not effect a cure. However, according to an abstract in the Cincinnati *Lancet-Clinic* of November 7, 1896, forty cases of ringworm of the scalp, in hospital out-patients, were treated by formalin applications. The preparation used was formalin in full forty-per-cent. strength, which was vigorously rubbed in with a brush or mop for ten minutes, the hair having been shaved round the patches. The application was repeated every other day on four occasions, and then entirely discontinued. Of the forty cases, only five required repainting from non-eradication of the disease. Microscopical examination was always made before commencing treatment, and the presence of the tricophyton verified.—*Therapeutic Gazette.*

